# Identification of Tissue miRNA Signatures for Pancreatic Ductal Adenocarcinoma

**DOI:** 10.3390/cancers16040824

**Published:** 2024-02-18

**Authors:** Carlo Caputo, Michela Falco, Anna Grimaldi, Angela Lombardi, Chiara Carmen Miceli, Mariateresa Cocule, Marco Montella, Luca Pompella, Giuseppe Tirino, Severo Campione, Chiara Tammaro, Antonio Cossu, Grazia Fenu Pintori, Margherita Maioli, Donatella Coradduzza, Giovanni Savarese, Antonio Fico, Alessandro Ottaiano, Giovanni Conzo, Madhura S. Tathode, Fortunato Ciardiello, Michele Caraglia, Ferdinando De Vita, Gabriella Misso

**Affiliations:** 1 Department of Precision Medicine, University of Campania “Luigi Vanvitelli”, Via L. De Crecchio 7, 80138 Naples, Italy; carlo.caputo@unicampania.it (C.C.); michela.falco@unicampania.it (M.F.); chiara.tammaro@unicampania.it (C.T.); madhura.tathode@unicampania.it (M.S.T.); fortunato.ciardiello@unicampania.it (F.C.); michele.caraglia@unicampania.it (M.C.); 2 Laboratory of Precision and Molecular Oncology, Institute of Genetic Research, Biogem Scarl, Contrada Camporeale, 83031 Ariano Irpino, Italy; 3 U.P. Cytometric and Mutational Diagnostics, AOU Policlinico, University of Campania “Luigi Vanvitelli”, Via Luciano Armanni 5, 83031 Naples, Italy; grim.anna@tiscali.it; 4 Department of Precision Medicine, Division of Medical Oncology, University of Campania “Luigi Vanvitelli”, Via L. De Crecchio 7, 80138 Naples, Italy; chiaracarmen.miceli@unicampania.it (C.C.M.); mariateresacocule1292@gmail.com (M.C.); luca.pompella@unicampania.it (L.P.); giuseppe.tirino@unicampania.it (G.T.); ferdinando.devita@unicampania.it (F.D.V.); 5 Department of Mental and Physical Health and Preventive Medicine, UOC Pathological Anatomy, University of Campania “Luigi Vanvitelli”, Via Luciano Armanni 5, 83031 Naples, Italy; marco.montella@unicampania.it; 6 Division of Anatomic Pathology, A.O.R.N. Antonio Cardarelli, 80131 Naples, Italy; severo.campione@aocardarelli.it; 7 Department of Medical, Surgical, and Experimental Sciences, University of Sassari, 07100 Sassari, Italy; cossu@uniss.it; 8 Department of Biomedical Sciences, University of Sassari, 07100 Sassari, Italy; gfenu@uniss.it (G.F.P.); mmaioli@uniss.it (M.M.); donatella.coradduzza@libero.it (D.C.); 9 Center for Developmental Biology and Reprogramming (CEDEBIOR), Department of Biomedical Sciences, University of Sassari, Viale San Pietro 43/B, 07100 Sassari, Italy; 10 AMES Center, Centro Polidiagnostico Strumentale SRL, Via Padre Carmine Fico 24, 80013 Casalnuovo Di Napoli, Italy; giovanni.savarese@centroames.it (G.S.); antonio.fico@centroames.it (A.F.); 11 Department of Abdominal Oncology, SSD-Innovative Therapies for Abdominal Metastases, Istituto Nazionale Tumori di Napoli, IRCCS “G. Pascale”, National Cancer Institute, 80131 Naples, Italy; a.ottaiano@istitutotumori.na.it; 12 Division of General, Oncological, Mini-Invasive and Obesity Surgery, University of Study of Campania “Luigi Vanvitelli”, 80138 Naples, Italy; giovanni.conzo@unicampania.it

**Keywords:** pancreatic ductal adenocarcinoma, microRNAs, biomarkers, prognosis, diagnosis

## Abstract

**Simple Summary:**

Pancreatic ductal adenocarcinoma (PDAC) is one of the most aggressive and deadliest cancers worldwide, with an overall survival rate of <10% at 5 years. For most patients with unresectable or recurrent PDAC, few therapeutic options are available, with limited efficacy. Furthermore, there is an almost complete absence of validated predictive factors. At present, microRNAs represent a promising diagnostic, prognostic, and predictive method for the clinical management of patients, avoiding inappropriate treatments.

**Abstract:**

Pancreatic ductal adenocarcinoma (PDAC), a neoplasm of the gastrointestinal tract, is the most common pancreatic malignancy (90%) and the fourth highest cause of cancer mortality worldwide. Surgery intervention is currently the only strategy able to offer an advantage in terms of overall survival, but prognosis remains poor even for operated patients. Therefore, the development of robust biomarkers for early diagnosis and prognostic stratification in clinical practice is urgently needed. In this work, we investigated deregulated microRNAs (miRNAs) in tissues from PDAC patients with high (G3) or low (G2) histological grade and with (N+) or without (N−) lymph node metastases. miRNA expression profiling was performed by a comprehensive PCR array and subsequent validation by RT-qPCR. The results showed a significant increase in miR-1-3p, miR-31-5p, and miR-205-5p expression in G3 compared to G2 patients (** *p* < 0.01; *** *p* < 0.001; *** *p* < 0.001). miR-518d-3p upregulation and miR-215-5p downregulation were observed in N+ compared to N− patients. A statistical analysis performed using OncomiR program showed the significant involvement (*p* < 0.05) of two miRNAs (miR-31 and miR-205) in the histological grade of PDAC patients. Also, an expression analysis in PDAC patients showed that miR-31 and miR-205 had the highest expression at grade 3 compared with normal and other tumor grades. Overall, survival plots confirmed that the overexpression of miR-31 and miR-205 was significantly correlated with decreased survival in TCGA PDAC clinical samples. A KEGG pathway analysis showed that all three miRNAs are involved in the regulation of multiple pathways, including the Hippo signaling, adherens junction and microRNAs in cancer, along with several target genes. Based on in silico analysis and experimental validation, our study suggests the potential role of miR-1-3p, miR-31-5p, and miR-205-5p as useful clinical biomarkers and putative therapeutic targets in PDAC, which should be further investigated to determine the specific molecular processes affected by their aberrant expression.

## 1. Introduction

Pancreas is a gland of the digestive and endocrine systems with both endocrine and exocrine functions [1]. Its key role is to maintain metabolic and energy homeostasis by producing and releasing various endocrine hormones and digestive enzymes [2]. Pancreatic dysfunctions can lead to several common diseases including diabetes [3], pancreatitis [4], and cancer. The latter is still a highly lethal gastrointestinal cancer, currently the fourth highest cause of cancer mortality worldwide, despite the rapid advances in modern medical technology and the significant improvements in survival rates of many other cancers [5,6,7]. The incidence of pancreatic cancer varies significantly between regions and populations, with the highest incidence rates in Europe, Northern America, and Australia/New Zealand, and the lowest in Middle Africa and South-Central Asia [8]. Based on 2012–2018 reports from Surveillance, Epidemiology, and End Results (SEER), the five-year survival rate is only 11.5%. The pancreatic ductal adenocarcinoma (PDAC) subtype accounts for most exocrine tumors and more than 90% of all pancreatic malignancies. PDACs are derived from epithelial cells of the pancreatic duct and appear gland-like due to their origin. To date, the causes of PDACs are still insufficiently clear, although behavioral factors and genetic susceptibility have been identified [9,10,11,12]. Many hypotheses about its pathogenesis have been proposed, and the evidence suggests that PDAC is the result of unpredictable mutations coupled with alterations in the environmental factors [13,14,15]. An average of 63 gene mutations (mostly point mutations) have been identified in each tumor; they can be organized into twelve known signal transduction mediators, whose alterations are fundamental to the oncogenic process [16]. Many of these mutations involve the KRAS oncogene (90–95% of cases), p16, and TP53 tumor suppressors, SMAD4\DPC4 [17,18], and, more rarely, ARID1A, MLL3, and TGFBR2 [19,20,21].

The early-stage diagnosis and treatment of PDAC are difficult, accounting for the poor clinical patient outcomes. There are no screening recommendations for pancreatic cancer, and there is no reliable screening test with adequate sensitivity and specificity that can have routine clinical use to screen the general population. Accordingly, PDACs are often diagnosed after the occurrence of lymph node and/or liver metastases, resulting in an estimated median survival as short as 4 months [22]. As for all other types of solid cancer, the TNM classification system provides helpful information, suggesting the most likely outcome and providing an estimate of life expectancy and the chance of a cure. The TNM system is based on factors related to the primary tumor (T), the number of involved lymph nodes (N), and the possibility of cancer spreading to other organs or parts of the body (M) [23]. Other factors are also important in determining prognosis. Tumor grade describes how closely abnormal cancer resembles normal tissue under a microscope. Specifically, Grade 1 (G1) refers to well-differentiated cancer cells, Grade 3 (G3) refers to poorly differentiated cancer cells, and Grade 2 (G2) falls somewhere in between, with moderately differentiated cancer cells. Low-grade cancers (G1) are less aggressive and tend to grow and spread more slowly than high-grade (G3) cancers, while Grade 3 pancreas cancers tend to have a poor prognosis compared to Grade 1 or 2 [24].

**Figure 1:**
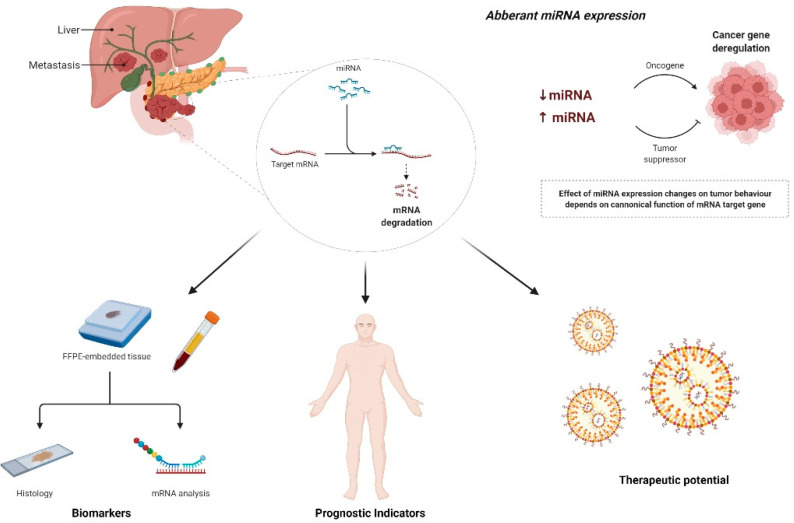
Research design.

Therapeutic options for PDAC, include surgical intervention, radiotherapy, chemotherapy treatment, target therapies, and combination therapies [25,26,27,28,29]. However, the best treatment choice for a single patient is still limited, and the clinical outcome not predictable even in the case of operable patients. In fact, progress in therapy and predictive insights are moderated by the inherent heterogeneity of PDAC at the molecular, pathological, and clinical tiers. This heterogeneity restricts the potential to enhance survival [30]. Efforts have been made to leverage genomic markers, molecular subcategories identified through extensive transcriptomic analysis, histological subgroups based on the World Health Organization (WHO) classification, and the trajectory of disease advancement for therapeutic and predictive purposes. Recently, the International Cancer Genome Consortium (ICGC) carried out a comprehensive genetic study involving 456 patients with PDAC. This study unveiled four distinct molecular subtypes within PDAC, each closely associated with specific histopathological features. These subtypes are as follows: (i) squamous, (ii) pancreatic progenitor, (iii) immunogenic, and (iv) aberrantly differentiated endocrine exocrine (ADEX) [31]. However, the results have fallen short of expectations [32,33,34]. This is primarily attributed to a few main factors, including the presence of numerous genes, often amounting to several hundred, within each subtype. This complexity poses challenges in conducting transcriptomic profiling across all patients, particularly when dealing with RNA sourced from paraffin-embedded tissues. Furthermore, the various endeavors in molecular subtyping have led to individual classification criteria for identifying subtype(s) linked to an unfavorable prognosis. As a result, a standardized gene panel for examination in clinical scenarios is lacking due to the absence of a consensus. These considerations push the scientific community to search for alternative molecular biomarkers that are easily determined and have a strong role in regulating the biological and metastatic potential of cancer cells. Among these markers, microRNAs (miRNAs) are attractive for several reasons [35,36,37,38]. miRNAs post-transcriptionally regulate gene expression in animals and plants by binding to the 3′ untranslated region (UTR), 5′ UTR, or/and coding regions of their corresponding target mRNAs in a sequence-specific manner. The targeting of mRNAs leads to the repression of protein synthesis using a mechanism that has yet to be fully determined. They are involved in crucial processes, such as cell division, cell proliferation, cell differentiation, programmed cell death (apoptosis), and blood vessel formation [39,40,41,42,43]. Their aberrant expression has been correlated with many human diseases, including cancer. As a single microRNA (miRNA) can influence the expression of a multitude of target genes, the notion arises that assembling a panel of miRNAs that regulate the expression of candidate genes specific to each PDAC subtype might offer a more robust and clinically significant signature. This approach is reinforced by the fact that previous investigations have convincingly showcased the frequent disruption of miRNA expression in various malignancies, including PDAC [44,45,46,47,48,49,50], and there is emerging evidence suggesting that miRNAs could function as crucial biomarkers in PDAC patients [51,52,53,54,55,56,57]. Moreover, the stability of miRNA expression across a range of clinical specimens enhances their appeal as reliable substitutes in situations in which gene expression analysis presents challenges. Additionally, the simplicity of detecting miRNAs in bodily fluids underscores their potential as biomarkers for liquid-biopsy-based assays, facilitating a smoother translation into clinical practice. In this study, we investigated a comprehensive miRNA expression profile in PDAC tissue samples from patients undergoing surgical resection, followed by a validation test employing quantitative reverse transcription PCR (RT-qPCR) to identify specific miRNA signatures reflecting two clinic-pathological features: PDAC histological grading (G3 grade versus G2 grade) and lymph node metastases (N+ versus N−). The research on miRNA signatures could also be useful in the development of new, reliable, and specific molecular biomarkers for the definition of PDAC prognosis, the prediction of lymph node metastases, the identification of new therapeutic candidates for either replacement therapy or miRNA inhibition, and for the identification of predictive factors of response to treatments (Figure 1).

## 2. Materials and Methods

### 2.1. Clinical Samples

Tissue samples were collected from PDAC patients enrolled at the University of Campania “Luigi Vanvitelli”, “Antonio Cardarelli” Hospital and Department of Anatomy and Pathological Histology at the University of Sassari. Informed consent was obtained from all patients. Across the three institutions, 58 clinical samples were enrolled and were assigned to 4 groups according to two specific clinical–pathological features: lymph node metastasis (N+ versus N− group) and tumor grading (G3 group versus G2 group). A total of 15 patients were assigned to the N− group, 38 patients to the N+ group, 23 patients to the G2 group and 29 patients to the G3 group. Patients’ clinical information is summarized in Table 1.

### 2.2. RNA Extraction

A formalin-fixed paraffin-embedded (FFPE) tissue block was selected for each patient. Four unstained FFPE tissue sections were cut at 10 μM each for RNA extraction. Total RNA, including miRNA, was extracted using miRNeasy FFPE Kit (Qiagen, Hilden, Germany), according to the manufacturer’s instructions, with the following modifications: the RNA extract was concentrated through a final elution in 30 μL of Nuclease Free-Water RNA. RNA purity and quantity were measured by a spectrophotometer, using the 260/280 ratio, with a NanoDrop ND-1000 (Thermo Scientific, Wilmington, NC, USA). RNA samples were stored at −80 °C until further processing.

### 2.3. Comprehensive PCR-Array-Based Screening Assay

Total RNA purified from the tissues of 18 patients was used for miRNA screening assay. The specimens of PDAC cohort were divided into four groups, i.e., 5 lymph node metastases positive (N+), 5 negative (N−), 5 grade G3, and 5 grade G2 tissue samples. For reverse transcription, an equal amount of RNA tissue from different patients was mixed into 4 pools according to two specific clinical–pathological features: lymph node metastasis (N+ versus N− group) and tumor grading (G3 group versus G2 group). N− group and G2 group were used as control groups. Starting from each RNA pool, cDNA was synthesized with Megaplex RT Primers, Human Pool A v2.1 (Applied Biosystems, Foster City, CA, USA), and TaqMan MicroRNA Reverse Transcription Kit (Applied Biosystems, CA, USA), according to the manufacturer’s instructions. miRNA expression profiling by TaqMan Array Human MicroRNA A Cards v2.0 (Applied Biosystems, CA, USA) and the Ct value determination were performed as previously described [58].

### 2.4. Real-Time Quantitative PCR

To confirm the findings obtained from PCR array screening, RT-qPCR analysis was performed with a TaqMan miRNA assay as a validation set. For the validation of miRNA candidates, tissue miRNA samples, originating from PDAC patients, were tested by qRT-PCR. cDNA synthesis was performed with TaqMan MicroRNA Reverse Transcription Kit (Applied Biosystems, CA, USA), according to the manufacturer’s manual. The expression levels of miRNA candidates were detected using TaqMan Universal PCR Master Mix (2X) (Applied Biosystems, CA, USA) under the control of the ViiA 7 real-time PCR system and QuantStudio 5 Dx Real-Time PCR System (Applied Biosystems, CA, USA). Comparative real-time PCR (RT-PCR) was performed in triplicate, including no template controls, and relative expression was calculated using the comparative cross-threshold (Ct) method. Cycle threshold (Ct) value was calculated using ViiA™ 7 Software v1.2 (Applied Biosystems) and QuantStudio 5 Dx (Applied Biosystems) with the threshold set to 0.2. Subsequently, for the normalization of target gene expression level, ΔCt was derived by the following formula: Ct of target gene—Ct of reference gene such as U6 snRNA (endogenous miRNA reference). ΔΔCt was calculated by the following formula: ΔCt of interest group—ΔCt of control group. Then, 2^−ΔΔCt^ was derived as a fold change (FC) of target gene expression.

### 2.5. In Silico miRNA Target Gene Prediction

Target genes were predicted for each differentially expressed miRNA (DEMs), identified using PCR-array-based screening assay, using three different online gene prediction tools, including TargetScanHuman Release 8.0 (https://www.targetscan.org/vert_80/, accessed on 1 March 2022) [59], DIANA microT-CDS v.5.0 (https://bio.tools/DIANA-microT, accessed on 1 March 2022) [60], and mirDB (https://mirdb.org/, accessed on 1 March 2022) [61]. A gene predicted by all the three-prediction program for miRNA is considered a predicted target. Furthermore, experimental target genes were identified using online TarBase v8 [62]. Out of the total predicted and experimental targets that were identified, target genes regulated by at least two out of three miRNAs from DEMs were considered for the downstream analysis.

### 2.6. Pathway and Gene Ontology Enrichment Analysis

Functional annotation, including a pathway and gene ontology analysis for miRNAs, was performed using online DIANA-mirPath v3.0 (https://dianalab.e-ce.uth.gr/html/mirpathv3/index.php?r=mirpath, accessed on 5 December 2022) [63]. The enrichment analysis method used for the mirPath analysis is Fisher’s Exact Test (hypergeometric distribution).

### 2.7. Correlation Study between miRNA Expression Levels and PDAC Patient’s Survival and Clinico-Pathological Features

To understand the involvement of three differentially expressed microRNAs (DEMs) in PDAC patients, an overall survival analysis was conducted using The Cancer Genome Atlas (TCGA) PDAC clinical datasets using online OncoLnc program (http://www.oncolnc.org/, accessed on 1 June 2022) [64]. Furthermore, the involvement of dysregulated miRNA with tumor progression and development is studied using TCGA clinical datasets using the online program OncomiR (https://oncomir.org/oncomir/index.html, accessed on 1 June 2022) [65], with a *p*-value significance cut-off of 0.05. OncomiR uses TCGA miRNA sequencing data from patients of different cancer types and conducts a statistical analysis to identify miRNAs associated with multiple clinical parameters, such as different clinical and pathological stages, different tumor grade, and different sex. Furthermore, tumor-grade-wise expression profile graphs of the chosen potential biomarker miRNA candidate in TCGA PDAC patients were derived from online web resource UALCAN [66].

### 2.8. Statistical Analysis

The construction of a clustered heatmap for microarray analysis was performed using heatmap.2 function of gplots package contained in statistical analysis tool R (version 3.4.3). To evaluate the difference in expression between the two groups, Student’s *t*-test was used to calculate *p*-value. Graphs were obtained using GraphPad Prism (version 7.00) and significant differences were determined at *p* ≤ 0.05 according to Student’s *t* test. ROC curve analysis, using R (version 3.4.3), was performed as previously described [67]. Data are expressed as the mean ± SD. *p* < 0.05 was considered statistically significant.

## 3. Results

### 3.1. Definition of Tissue miRNA Signature

**Figure 2:**
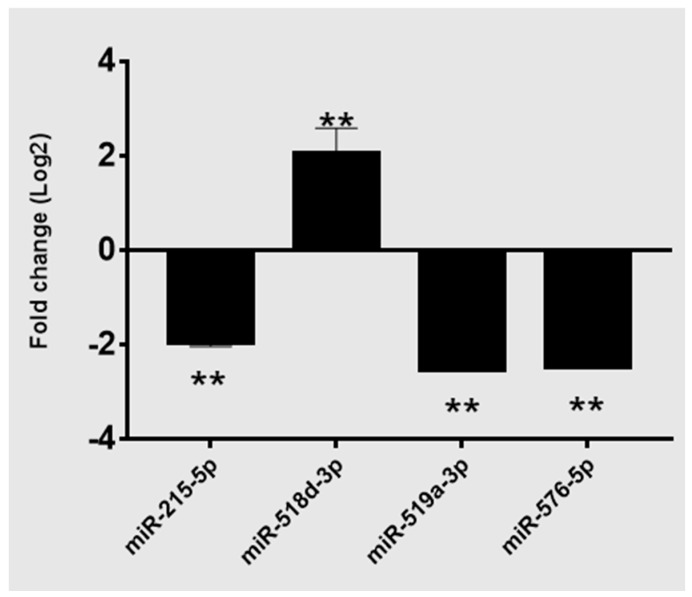
The expression level of the most significantly dysregulated miRNAs in N+ vs. N− comparison. ** *p* < 0.05.

We performed a comprehensive PCR-array-based screening, as described in Section 2, to determine miRNA signatures in PDAC tissues. The clinical parameters of enrolled patients are summarized in (Table 1). A high-throughput PCR array analysis was performed on total RNA extracted from a cohort of 18 PDAC patients, divided into four groups, as mentioned in the Materials and Methods. The statistical analysis of the obtained data set allowed us to evaluate the significant difference in expression among the groups. Three inclusion criteria were set—(1) mean Ct < 32.0; (2) mean fold change (Log2) < −1.8 or >1.8; (3) *p*-value < 0.05—in order to select reliable dysregulated miRNAs. According to the expression pattern, seven miRNAs were statistically deregulated (two up- and five downregulated miRNAs) between N+ and N− cancers (Table 2). Among them, upregulated miR-518d-3p and downregulated miRNAs miR-215-5p, miR519a-3p, and miR576-5p were the most significant (Figure 2).

**Figure 3:**
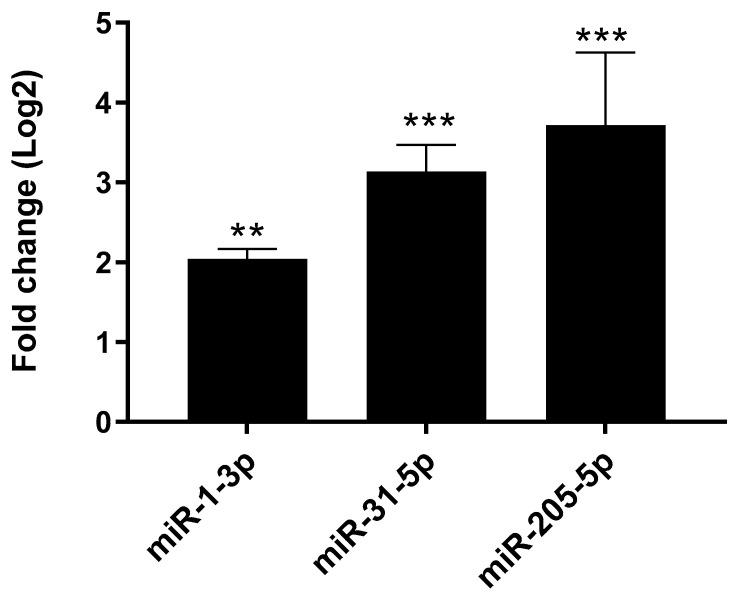
The expression level of the most significantly dysregulated miRNAs in G3 vs. G2 comparison. The *p*-value was calculated by *t*-test ** *p* < 0.05; *** *p* < 0.005.

Comparing the G3 with the G2 group, 11 differently expressed miRNAs were observed, associated with the most advanced grading (G3), as follows: 8 up-regulated miRNAs and 3 down-regulated miRNAs (Table 3). miR-1-3-p, miR-31-5p, and mR-205-5p were significantly upregulated in the G3 group compared to the G2 group (Figure 3).

These results showed the significant modulation of several miRNAs in PDAC tissues, thus suggesting their implication in biological processes such as carcinogenesis and malignant metastasis, as well as their potential role in both neoplasm diagnosis and treatment.

### 3.2. Validation Study of Candidate miRNAs in PDAC Tissues

**Figure 4:**
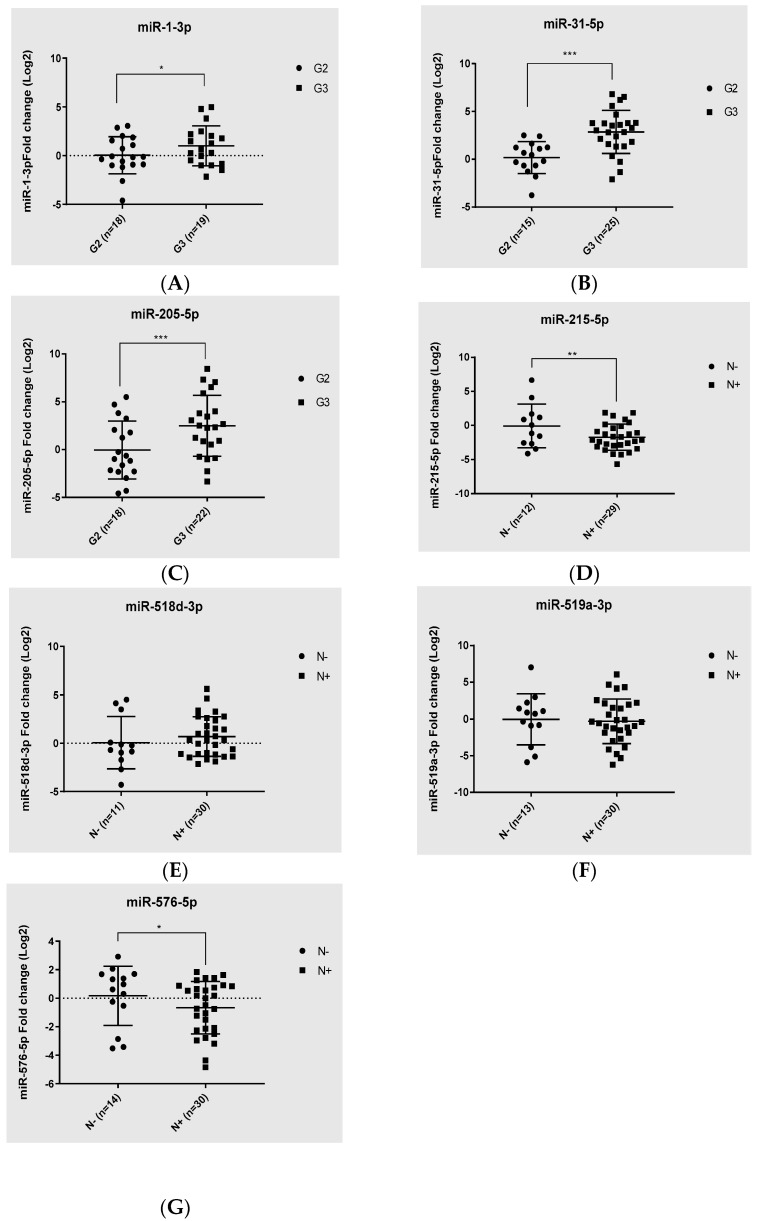
Validation of candidate miRNAs expression in PDAC Tissues. The expression levels of (**A**) miR-1-3p, (**B**) miR-31-5p, (**C**) miR-205-5p, (**D**) miR-215-5p, (**E**) miR-518d-3p, (**F**) miR-519a-3p, and (**G**) miR-576-5p were validated in PDAC tissues and the paired control ones using RT-qPCR. For the normalization, U6 snRNA was used as the endogenous control. Each sample was run in triplicate. Error bars show mean ± SD. A *t* test was used for the calculation of *p* values. *** *p* < 0.005, ** *p* < 0.05, * *p* < 0.05.

To confirm the findings obtained from the PCR array screening, we performed an RT-qPCR analysis with a TaqMan miRNA assay as a validation set on the entire population of enrolled patients. The results confirmed a significant upregulation of miR-1-3p, miR-31-5p, and miR-205-5p in G3 pts compared to G2 (Figure 4A–C). On the other hand, only the significant upregulation of miR-518d-3p and the downregulation of miR-215-5p were confirmed for miRNAs deregulated in N+ versus N− patients (Figure 4D–G).

### 3.3. ROC Curve Analysis

**Figure 5:**
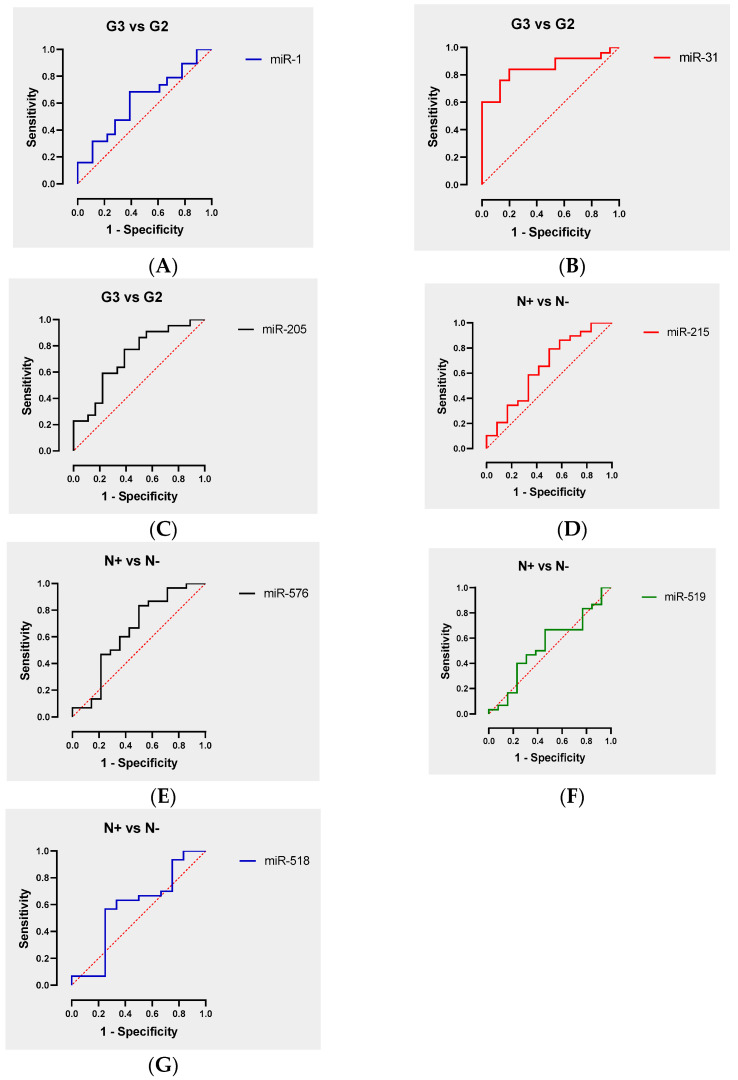
ROC curve analysis of each validated miRNA for an evaluation of biomarker potential. (**A**) miR-1-3p in G3 tissues compared to the G2 tissues (sensibility = 0.63; specificity = 0.61; AUC = 0.62, *p*-value: 0.2242); (**B**) miR-31-5p in G3 tissues compared to the G2 tissues (sensibility = 0.88; specificity = 0.47; AUC = 0.85; *p*-value: 0.0003); (**C**) miR-205-5p in G3 tissues compared to the G2 tissues (sensibility = 0.77; specificity = 0.61; AUC = 0.72; *p*-value: 0.0194). (**D**) miR-215-5p in N+ tissues compared to the N− tissues (sensibility = 0.79; specificity = 0.42; AUC = 0.65, *p*-value: 0.1439); (**E**) miR-576-5p in N+ tissues compared to the N− tissues (sensibility = 0.57; specificity = 0.64; AUC = 0.64, *p*-value: 0.1306); (**F**) miR-519a-3p in N+ tissues compared to the N− tissues (sensibility = 0.63; specificity = 0.54; AUC = 0.54, *p*-value: 0.6916); (**G**) miR-518d-3p in N+ tissues compared to the N− tissues (sensibility = 0.57; specificity = 0.67; AUC = 0.58; *p*-value: 0.4036). The red dashed line represents a classifier with the random performance level.

To estimate the power of each validated miRNA in detecting PDAC, we used the receiver operating characteristic (ROC) curve analysis, which provides a potent tool to select optimal diagnostic markers. The results showed the ability of each miRNA to reveal pancreatic cancer at G3 or with the involvement of lymph nodal metastases. Based on the sensitivity, specificity, and area under the curve (AUC), we assessed a good predictive performance for miR-31-5p and miR-205-5p as a molecular marker for the diagnosis of PDAC patients with G3 tumor grade. Instead, from the comparison between N+ patients and N− patients, ROC curves did not show significant predictive value for the analyzed miRNAs, likely due to the low number of analyzed samples (Figure 5).

### 3.4. Correlation Study between miRNA Expression Levels and PDAC Patients’ Survival and Clinico-Pathological Features

**Figure 6:**
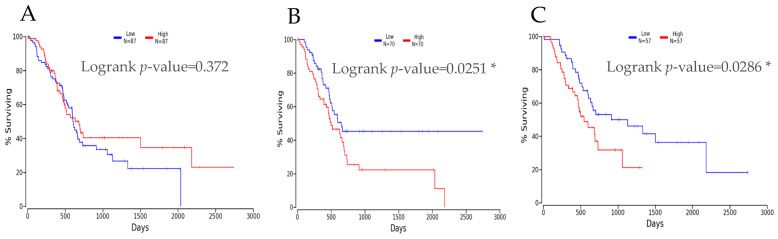
Kaplan–Meier survival analysis. Kaplan–Meier survival analysis of PDAC patients expressing low or high levels of miR-1-3p (**A**), miR-31-5p (**B**), and miR-205-5p (**C**). *: hsa-miR-31-5p and has-miR-205-5p were significantly associated with PDAC.

**Figure 7:**
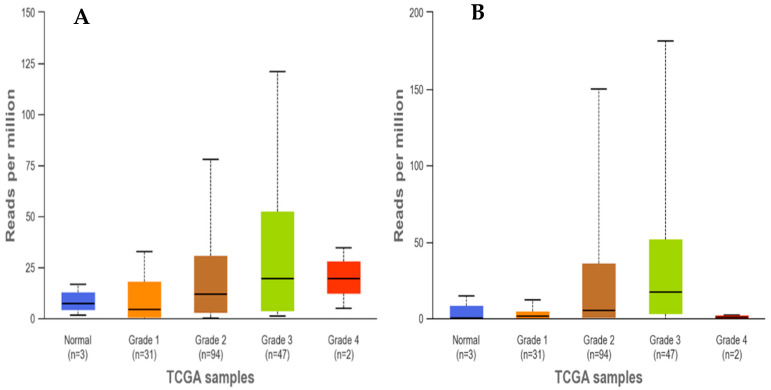
UALCAN analysis of DEMs expression in different PDAC grades. Grade-wise expression of miR-31 (**A**) and miR-205 (**B**), derived using UALCAN program. Analysis showed the highest expression of these two miRNAs in grade 3, in comparison to normal and grades 1, 2, and 4.

The involvement of differentially expressed miRNAs (DEMs) associated with PDAC was studied using Kaplan–Meier overall survival plots, derived using online OncoLnc program [60]. OncoLnc derives survival correlations using TCGA PDAC RNA-Seq expression data. Kaplan–Meier plots for DEMs, derived using OncoLnc, are depicted in Figure 6. An overall survival analysis showed a significant involvement of miR-205-5p and miR-31-5p in PDAC (with a significant log-rank *p*-value < 0.05). It was also observed that the overexpression of miR-205-5p and miR-31-5p was significantly associated with decreased survival in PDAC patients. On the other hand, the overexpression of miR-1-3p was associated with increased survival in TCGA clinical datasets. This corroborates the finding of a significant association between these two miRNAs and PDAC occurrence, suggesting their potential role as prognostic markers in PDAC patients. Furthermore, OncomiR analysis showed a significant correlation between miR-31-5p expression and T, N, and M status, and confirmed the significant association between miR-31-5p and miR-205-5p and histological grade in PDAC (*p*-value ≤ 0.05). Detailed results of the OncomiR analysis are represented in Table 4. Additionally, an analysis of the extent of DEM expression using UALCAN showed an increased expression of miR-31 and miR-205, specifically in grade 3, in comparison to normal and grade 1, 2, and 4 TCGA PDAC patients (Figure 7).

### 3.5. Identification of Target Genes Associated with Differentially Expressed miRNA Signatures

A bioinformatic analysis was performed with the aim of deepening the precise molecular context in which the oncogenic activity of DEMs occurs. For this investigation, we included all three DEMs correlated with tumor grading. In fact, although no significant correlations were found between miR-1-3p overexpression and PDAC pathological parameters, nor was any significant predictive performance identified for this miRNA, its significant upregulation emerged in both the discovery and the validation phase, represent a clear indication that miR-1-3p could exert a non-negligible role, equally prominent to that of miR-31-5p and mir-205-5p, in the molecular landscape of PDAC progression. Therefore, we performed a broad evaluation of DEMs’ targets and inherent pathways to identify possible commonly regulated genes as key mediators of the underlying mechanisms of action.

**Figure 8:**
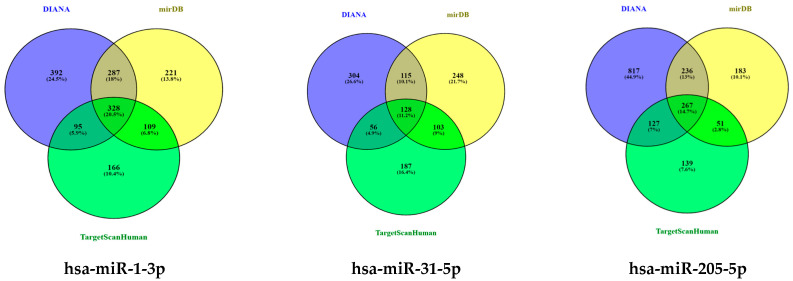
Venn diagram of cross-regulated DEMs’ targets. The count of target genes predicted for miR-1-3p, miR-31-5p, and miR-205-5p dysregulated in G3 versus G2 in pancreatic adenocarcinoma using three different gene prediction tools.

The results of a target gene prediction analysis using three different algorithms are described in Figure 8. Only those targets which were predicted by all the three tools were considered. A total of 328, 128, and 267 predicted targets were identified for miR-1-3p, miR-31-5p, and miR-205-5p, respectively. Also, the experimental gene targets identified for each miRNA are described in summary statistics (Table 5). A detailed list of predicted and experimental target genes for DEMs is described in Supplementary Spreadsheet S1.

Specifically, we identified CLOCK, MAP3K, RSBN1, PAX5, SPRED1, CBL, HIAT1, DICER1, and PIK3CA as potential common targets regulated by both miRNAs (miR-1-3p and miR-31-5p) expressed in tissues with an advanced degree of differentiation. These genes are known to regulate multiple cancer-related processes, such as angiogenesis, apoptosis, proliferation, EMT, migration, and invasion.

### 3.6. Pathway and Gene Ontology Analysis of DEMs

**Figure 9:**
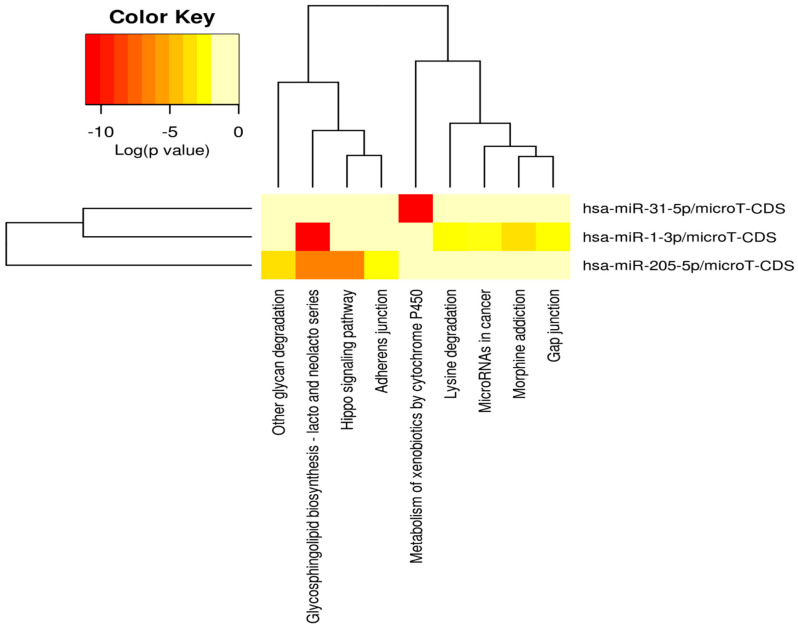
Heatmap of significant KEGG pathways predicted by DIANA-mirPath v3.0, regulated by three differentially expressed miRNAs. The color code represents log (*p*-value), where the most pathway is colored in red.

**Figure 10:**
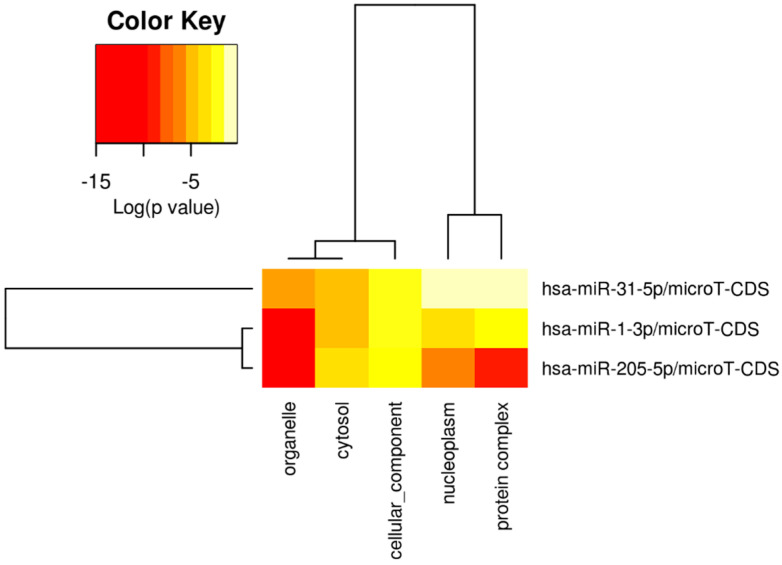
Heatmap of significant gene ontologies: cellular components predicted by DIANA-mirPath v3.0, regulated by three differentially expressed miRNAs. The color code represents log (*p*-value), where the most significant gene ontology term is colored in red.

**Figure 11:**
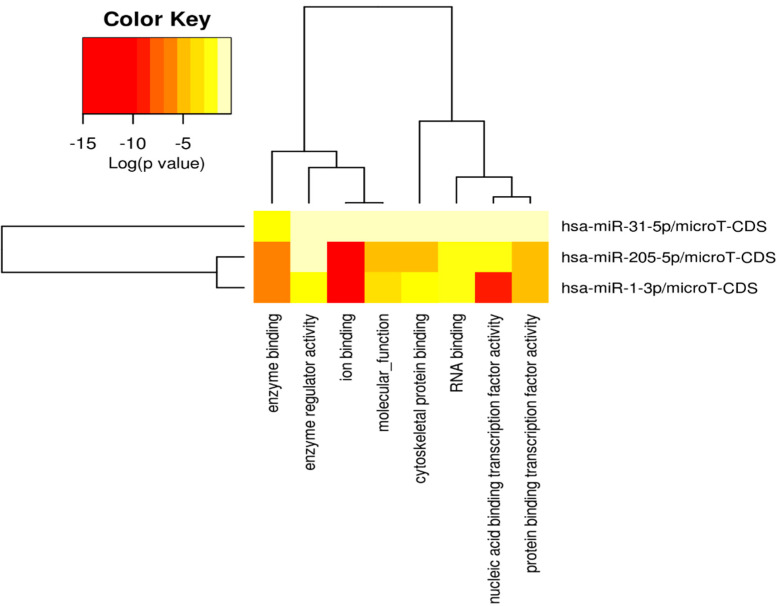
Heatmap of significant gene ontologies: molecular functions predicted by DIANA-mirPath v3.0, regulated by three differentially expressed miRNAs. The color code represents log (*p*-value), where the most significant gene ontology term is colored in red.

**Figure 12:**
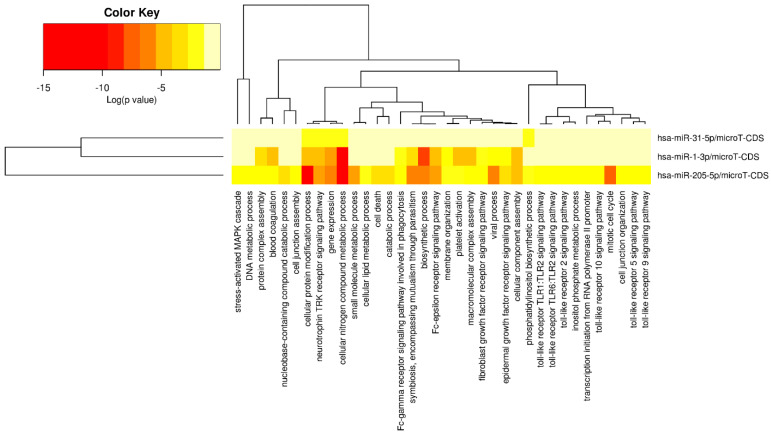
Heatmap of significant gene ontologies: biological processes predicted by DIANA-mirPath v3.0, regulated by three differentially expressed miRNAs. The color code represents log (*p*-value), where the most significant gene ontology term is colored in red.

A function enrichment analysis including the pathways and gene ontology terms regulated by DEMs was carried out using DIANA-mirPath v3.0 [63] with *p*-value threshold of 0.05. The KEGG pathways regulated by DEMs, along with the different target genes, are showed in Figure 9. From the list of identified pathways, the ones associated with the predicted and experimental target genes for input DEMs were identified. Further analysis showed that pathways including Hippo Signaling, Adherens junction, MicroRNAs in cancer, and the bacterial invasion of epithelial cells were regulated by all the three DEMs, i.e., miR-1-3p, miR-31-5p, and miR-205-5p. Also, gene ontology terms, including molecular function, cellular component, and biological process, were identified for DEMs (Figure 9, Figure 10, Figure 11 and Figure 12). Among the cellular components, the most significantly enriched were “organelle” and “cytosol” (Figure 10), whereas “ion binding” and “enzyme binding” were the molecular functions with the largest lists of associated genes (Figure 11). Concerning the biological processes, we found significant GO terms, including “cellular nitrogen compound metabolic process”, “TRK receptor signaling pathway”, and “mitotic cell cycle”, to be the most enriched (Figure 12). Furthermore, we found multiple KEGG-significant pathways that were enriched by DEMs, including “microRNAs in cancer”, “Hippo signaling pathways”, and “Glycosphingolipid biosynthesis” (Figure 9).

## 4. Discussion

Pancreatic cancer is one of the leading causes of cancer mortality in developed countries and one of the most lethal malignant gastrointestinal neoplasms across the world [6]. Based on GLOBOCAN 2020 estimates, pancreatic cancer accounts for more than 495,773 new diagnoses and 466,003 deaths yearly worldwide, ranking as the seventh leading cause of cancer death in both sexes [7,8]. The past two decades have seen a doubling in the annual number of diagnosed pancreatic cancers worldwide. To date, the causes of pancreatic cancer are still insufficiently known, although certain risk factors have been identified, such as smoking, obesity, genetics, diabetes, diet, and inactivity [9,12]. The two main histological types of pancreatic cancer are adenocarcinoma (more than 90% of cases), and pancreatic endocrine tumors (less than 5% of all cases). Despite numerous advances in pancreatic cancer therapy, radical surgery is currently the only option that is able to offer a concrete advantage in terms of overall survival, but it is still an evolving field [26,30]. However, due to the absence of specific symptoms and reliable markers, PDAC is usually diagnosed in advanced stages, resulting in delayed treatment, a high frequency of tumor recurrence, metastasis, and a worse prognosis. Therefore, alternative therapeutic approaches are needed. Among all the treatments available for PDAC, the options for locally advanced inoperable and/or metastatic disease usually include chemotherapy and targeted therapies aimed at exploiting specific genetic or molecular targets on both tumor and stromal cells. However, PDAC presents challenges in treatment selection and predicting outcomes due to its molecular and clinical diversity. Efforts to improve therapy and prediction are hindered by the complex variability in the disease’s genetics, pathology, and clinical behavior. Various approaches, including genomic markers, transcriptomic analyses, histological classifications, and disease progression patterns, have been used in an attempt to guide treatment decisions. A genetic study involving 456 PDAC patients identified four distinct molecular subtypes (squamous, pancreatic progenitor, immunogenic, and aberrantly differentiated endocrine exocrine), each tied to specific histopathological features. However, these efforts have not met expectations, partly due to the complexity of genes within each subtype and the challenges in profiling patient tissues. The different classification criteria in these studies also contribute to the lack of a standardized gene panel for clinical use.

As a result, researchers are exploring alternative molecular biomarkers with potential roles in regulating cancer cell biology and metastasis, with microRNAs (miRNAs) standing out as promising candidates. miRNA identification in the bloodstream or other biological fluids, as well as in tissue samples, has generated great interest for their potential use as clinical biomarkers. Circulating miRNAs are a new class of gene regulator, whose role in cancer onset and progression has been deepened, opening new opportunities for therapeutic application. They belong to the family of small non-coding RNA molecules of ~24 nt in length that can inhibit mRNA translation and/or negatively regulate its stability. In the last years, an increasing number of dysregulated miRNAs in plasma or serum and tissue have been considered a novel hallmark of cancer. A large body of evidence showed aberrant miRNA regulation in PDAC tissues, demonstrating a strong relationship between their expression levels and the main processes underlying tumor initiation and progression, such as proliferation, migration, invasion, metastasis, tumor infiltration, and disease relapse. Several reports have delineated a possible oncogenic or tumor-suppressive role for several miRNAs in PDAC pathogenesis; however, the molecular mechanism is still unclear and further insights are required for their use as diagnostic, prognostic, and therapeutic tools.

In this scenario, the objective of the present work was to determine a miRNA signature for the definition of PDAC risk stratification. To achieve this aim, we performed a comprehensive PCR-array-based screening assay on total RNA extracted from a cohort of eighteen PDAC patients, divided into four groups: five lymph node metastases positive (N+), five negatives (N−), five grade G3, and five grade G2 tissue samples. The global miRNA expression profile allowed us to detect seven dysregulated miRNAs in the N+ group compared to the N− group. Among them, we observed the significant upregulation of miR-138-5p and miR-518d-3p, and significant downregulation of miR-215-5p, miR-519a-3p, miR-522-3p, miR-576-5p, and miR-147-5p (Table 2). Moreover, in the comparison between the G3 group and G2 group, we detected eleven dysregulated miRNAs and, specifically, a significant upregulation of miR-1-3p, miR-31-5p, miR-133a-3p, miR-137-3p, miR-187-3p, miR-205-5p, miR-215-5p, and miR-490-3p, and significant downregulation of miR-380-3p, miR-451-5p, and miR-517b-5p (Table 3). Subsequently, based on statistical significance, we focused on three upregulated miRNAs—miR-1-3p, miR-31-5p, and miR-205-5p—as possible markers candidates for validation. The RT-qPCR analysis, performed on fifty-eight enrolled patients, confirmed the significant upregulation of miR-1-3p, miR-31-5p, and miR-205-5p in G3 pts compared to G2 (Figure 4A–C). On the other hand, only the significant upregulation of miR-518d-3p and the downregulation of miR-215-5p was confirmed for miRNAs deregulated in N+ versus N− patients (Figure 4D,E). These data are partly supported by a bioinformatic analysis showing the correlation between the expression levels of upregulated miRNAs and patients’ survival. For miR-1-3p no correlation was found. On the other hand, both miR-31-5p and miR-205-5p showed a significant correlation between high expression levels and decreased survival (Figure 6B,C). The latter findings agree with our experimental results, highlighting the upregulation of the two miRNAs in the G3 group (with poor prognosis) compared to the G2 group. Moreover, miR-31-5p was also significantly correlated with T, N, and M stage, and especially with histologic grade. In addition, a significant correlation with histologic grade was found for miR-205-5p (Figure 7 and Table 4).

To obtain a better understanding of the molecular pathways regulated by miR-1-3p and miR-31-5p, we carried out in silico prediction assays to identify potential miRNA target genes, interrogating three different algorithms of miRNA target prediction (Figure 8 and Table 5). The target genes predicted by all three tools were selected for the subsequent in silico detection of commonly regulated genes by both miRNAs, identifying the following commonly regulated genes: CLOCK, MAP3K1, RSBN1, PAX5, SPRED1, CBL, HIAT1, DICER1, and PIK3C2A. MAP3K1 is a member of the mitogen-activated protein kinase kinase kinase (MAP3K) family of serine/threonine kinases. The full-length form regulates cell migration and contributes to pro-survival signaling, while the cleaved form promotes apoptosis. The critical function of MAP3K1 in cell fate decisions suggests that it may be a target for deregulation in cancer [68]. SPRED1 is an important negative regulator of the Ras-MEK-ERK signaling pathway [69]. C-Cbl is a key negative regulator of cell signaling. Its downregulation in gastric carcinoma (GC) tissues plays an important role in GC development and progression. In addition, c-Cbl expression levels correlate with GC subtype, histological type, histological differentiation, and lymph node metastasis [70]. PAX5 is a member of the paired box (PAX) family of transcription factors containing a highly conserved DNA-binding domain, known as the paired box. The PAX proteins are important regulators in early cancer development, differentiation, migration, invasion, and proliferation. Therefore, its aberrant alterations are thought to contribute to neoplastic transformation [71,72]. PAX5 is extensively studied in lymphoma and lymphocytic leukemia, working as an oncogene related to developmental defects in B cells [73]. Dicer is an enzyme involved in the process of biogenesis and maturation of miRNAs. Its overexpression is positively correlated with advanced PDAC and acquired resistance to gemcitabine [74]. The function enrichment analysis shown in Figure 9 suggests that the Hippo signaling pathway, adherens junction, and microRNAs in cancer are regulated by all three miRNAs. The list of genes regulated by miRNAs was analyzed with the DIANA-mirPath v3.0 for pathway and molecular function enrichment. The main molecular functions that were identified included “ion binding” and “enzyme binding” (Figure 11). Instead, among the biological processes, the most significantly enriched included “cellular nitogen compound metabolic process”, “TRK receptor signaling pathway”, and “mitotic cell cycle” (Figure 12). Bott et al. demonstrated that GLUL-mediated glutamine synthesis plays a critical role in converging the TCA cycle and nitrogen metabolism to promote nitrogen-dependent anabolic processes in pancreatic cancer, and they observed a positive correlation between GLUL expression and human pancreatic cancer progression [75]. Likewise, preclinical studies showed that the inhibition of TRK receptor signaling could affect pancreatic cancer cell invasion, survival, and chemotherapy response [76]. Finally, there are different genes involved in the cell cycle pathway, whose mutations can interfere with apoptosis, proliferation, and migration mechanisms, thus correlating with PDAC progression and prognosis [77,78,79].

Collectively, our findings, based on both experimental and bioinformatics results, suggest the potential capacity of miR-1-3p, miR-31-5p, and miR-205-5p as useful clinical biomarkers, which are able to discriminate PDAC risk. Finally, they are potential therapeutic targets in PDAC, having commonly regulated genes that are strongly involved in cancer biology regulation.

## 5. Conclusions

Pancreatic ductal adenocarcinoma (PDAC) is a major cause of cancer-associated mortality. New effective therapeutic and diagnostic approaches are still lacking, and the prognosis remains poor, even for resectable patients. Furthermore, there is an almost complete absence of validated predictive markers for risk stratification. This gap needs to be urgently reduced so that patients’ survival and quality of life can be improved. Currently, prevention or early diagnosis at a curable stage is very difficult; patients exhibit symptoms only later, and tumors do not show sensitive and specific markers to aid in detection. For more effective clinical management, robust biomarkers for early diagnosis and prognostic stratification are needed in clinical practice, especially in the context of neoadjuvant and adjuvant settings. The advancements in molecular and genetic technologies allow for the identification of molecular factors endowed with a diagnostic and/or prognostic role, showing their biomedical potential for application in personalized medicine and the treatment monitoring of PDAC patients. Many studies revealed a pivotal role of non-coding RNAs, mainly miRNAs, in cancer initiation and progression, as well as in chemo-resistance mechanisms, suggesting their use as clinical biomarkers. Our study highlighted, for PDAC cancer patients, possible miRNA signatures reflecting two clinical and pathological characteristics: node metastases’ occurrence and tumor grading. In tissue samples from PDAC patients with advanced grading (G3), the significant upregulation of three miRNAs—miR-1-3p, miR-31-5p, and miR-205-5p—was observed and validated. In addition, through bioinformatic studies, the expression of two of these miRNAs was significantly correlated with survival, while all three miRNAs were significantly correlated with four clinical parameters—TNM status and histological grade—supporting our experimental evidence. Taken together, these data suggest their promising function as prognostic biomarkers. Using bioinformatic analyses, we also predicted a series of possible target genes that are likely responsible for the downstream oncogenic effects of both miR-1-3p and miR-31-5p. In conclusion, these data provide a strong rationale for the further investigation of miRNA involvement in PDAC onset and progression.

## Figures and Tables

**Table 1:** Clinical information for all enrolled PDAC patients.

Characteristics		n. (%)
Age (year)	≥60	15 (25.4)
	≤60	44 (74.6)
Sex	Male	27 (45.8)
	Female	32 (54.2)
Tumor grading	G3	33 (57.9)
	G2	24 (42.1)
LMN	N+	40 (70.2)
	N0	17 (29.8)

**Table 2:** Significantly deregulated miRNAs among PDAC tissues in N+ pts compared to N− pts.

miRNA	Regulation	Fold Change (Log2)	S.D.	*p*-Value
miR-138-5p	Up	2.820362	0.896241	0.004896
miR-215-5p	Down	−1.94699	0.076042	0.018501
miR-518d-3p	Up	2.045313	0.552661	0.013743
miR-519a-3p	Down	−2.51094	0.043237	0.010099
miR-522-3p	Down	−3.23667	0.039017	0.013339
miR-576-5p	Down	−2.45585	0.014635	0.026540
miR-147-5p	Down	−2.0902	0.112546	0.016185

**Table 3:** Significantly deregulated miRNAs among PDAC tissues in G3 pts compared to G2 pts.

miRNA	Regulation	Fold Change (Log2)	S.D.	*p*-Value
miR-1-3p	Up	2.038468	0.129115	0.019909
miR-31-5p	Up	3.134220	0.335566	0.000190
miR-133a-3p	Up	1.890646	0.243735	0.004635
miR-137-3p	Up	2.051514	0.123922	0.000221
miR-187-3p	Up	5.908869	1.38753	0.010564
miR-205-5p	Up	3.717156	0.910863	0.001044
miR-215-5p	Up	3.113365	1.570617	0.013060
miR-380-3p	Down	−3.31882	0.052619	0.066883
miR-451-5p	Down	−2.64865	0.018094	0.000968
miR-490-3p	Up	2.672909	0.490397	0.022821
miR-517b-5p	Down	−3.94387	0.029408	0.013197

**Table 4:** OncomiR analysis of DEMs’ correlation with PDAC grade. Results for statistical ANOVA and Multivariate Cox analysis performed by OncomiR to evaluate the miRNAs closely associated with tumor grade in TCGA PDAC datasets (*p*-value threshold used: <0.05). Out of three shortlisted DEMs, miR-31-5p and miR-205-5p were found to be significantly associated with tumor grade, and miR-31-5p was also found to be significantly associated with T, N, and M status in PDAC clinical patients.

miRNA Name	Cancer Abbreviation	Clinical Parameter	ANOVA *p*-Value	ANOVA FDR	Multivariate Log Rank *p*-Value	Multivariate Log Rank FDR
**hsa-miR-31-5p**	PDAC	Histologic Grade	0.036400	0.203000	0.036432	0.203282885
**hsa-miR-31-5p**	PDAC	Pathologic N Status	0.027600	0.297000	0.027573	0.297193659
**hsa-miR-31-5p**	PDAC	Pathologic Stage	0.037100	0.203000	0.037058	0.203296320
**hsa-miR-31-5p**	PDAC	Pathologic M Status	0.011100	0.089500	0.011058	0.089510893
**hsa-miR-205-5p**	PDAC	Histologic Grade	1.23 × 10^−4^	1.45 × 10^−3^	0.220247	0.502556231

**Table 5:** Summary statistics of predicted and experimental target genes for DEMs in G3 vs. G2 PDAC patient samples.

DE miRNA	DIANA-microT-CDS	miRDB	TargetScan_Human_v8.0	Count of Common Predicted Gene Targets (Predicted by All 3 Tools)	Count of Experimental Gene Targets (TarBase)
hsa-miR-1-3p	1121	945	698	328	117
hsa-miR-31-5p	603	594	474	128	19
hsa-miR-205-5p	1448	737	585	267	44

## Data Availability

The data presented in this study are available in this article (and Supplementary Materials).

## Supplementary Materials

The following supporting information can be downloaded at: https://zenodo.org/records/10443198, accessed on 16 February 2024. Spreadsheet S1: DEMs_TargetGenes_G3vsG2.xlsx.
